# Editorial: Flavonoids: From Biosynthesis and Metabolism to Health Benefits

**DOI:** 10.3389/fpls.2021.727043

**Published:** 2021-09-28

**Authors:** M. Carmen González-Mas, M. Amparo Blázquez, M. Pilar López-Gresa, Pedro Mena, Cristina García-Viguera

**Affiliations:** ^1^Department of Pharmacology, Faculty of Pharmacy, University of Valencia, Valencia, Spain; ^2^Institute for Plant Molecular and Cellular Biology, Spanish National Research Council (CSIC)-Polytechnic University of Valencia, Valencia, Spain; ^3^Human Nutrition Unit, Department of Food & Drug, University of Parma, Parma, Italy; ^4^Phytochemistry and Healthy Foods Laboratory, Department of Food Science and Technology, Centro de Edafología y Biología Aplicada del Segura-Consejo Superior de Investigaciones Científicas (CEBAS–CSIC), Murcia, Spain

**Keywords:** anthocyanin content, isoflavones, catechin galloylation, pinostrobin pharmacokinetics, xanthohumol, hydroxysafflor Yellow A, vitexin, formononetin

Flavonoids are a wide class of polyphenols with multiple biological activities. Unfortunately, many mechanisms of action that explain these activities are still unknown or there are insufficient data on their pharmacokinetics (Figure 1). Moreover, there are even undiscovered aspects about flavonoid biosynthesis. Expanding the knowledge on these topics will allow to regulate their biosynthesis, discover efficient preventive/therapeutic flavonoids for patients with certain diseases or design better clinical trials that help confirm their preventive/therapeutic usefulness. This Research Topic shows recent studies advancing along these lines.

**Figure 1:**
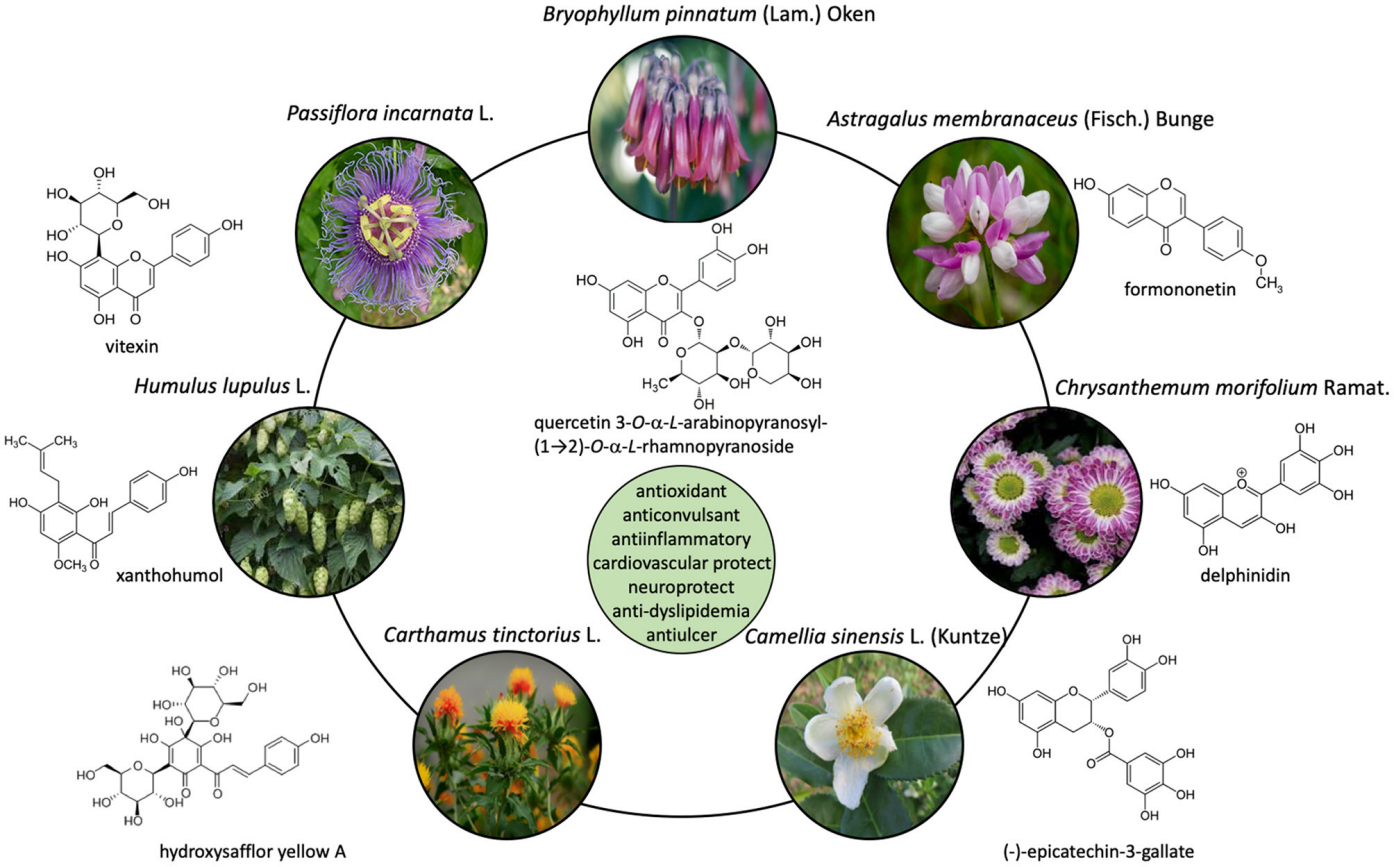
Structures of flavonoids isolated in several plant species.

Regarding flavonoid biosynthesis, this Research Topic presents a study about the catechin galloylation mechanism in tea plants. Serine carboxypeptidase-like acyltransferases (SCPL) are involved in this mechanism, but their roles have not been clarified so far. In this Research Topic, the tea genome-wide analysis of SCPL gene family is shown (Ahmad et al.), grouping several SCPLs into class IA (enzymes with an acylation function). Three SCPL-IA enzymes (CsSCPL11-IA, CsSCPL13-IA, CsSCPL14-IA) with galloylation activity toward catechins were assessed using recombinant enzymes. The expression levels of these SCPL-IA genes coincided with the galloylated catechin accumulation in tea plants, and their recombinant enzymes also displayed β-glucogallin:catechin galloyl acyltransferase activity. For the first time, genes encoding glucogallin:catechin galloyl acyltransferases with an active function in the galloylated catechin biosynthesis in tea plants have been identified. These results will help to understand the biosynthesis of these galloylated metabolites and the physiological roles of (–)-epicatechin-3-gallate, and (–)-epigallocatechin-3-gallate in tea plants.

The Research Topic also gives information about anthocyanin content in fruits and their biosynthesis regulation. Zeng et al. studied differences of sensory properties and proanthocyanidin and anthocyanin contents, among other phenolics, in rabbiteye blueberry “Brightwell” (*Vaccinium ashei* cv. “Brightwell”) fruits. This research showed that many blueberry sensory aspects (bitterness, umami, and sweetness) were positively correlated with total flavonoid, total phenol, proanthocyanidin, and anthocyanin contents. Blueberry plants grown at high altitude locations presented a high content of these compounds along with high scores for sweetness. These results suggested cultivating blueberry at high altitude may produce fruit that not only possess pronounced beneficial compounds but also good taste. In another work, Zhang B. et al. investigated the anthocyanin accumulation regulation in apple peel. This study focused on microRNAs, which participate in plant development by regulating gene expression at the posttranscriptional level. This research evidenced the increase expression level of the microRNA mdm-miR828 in the late apple fruit coloration stage, not in the rapid fruit coloration period, inhibiting anthocyanin synthesis in apple. Thus, mdm-miR828 is involved in a feedback regulatory mechanism associated with anthocyanin biosynthesis. In addition, it was also detected that mdm-miR828 inhibits anthocyanin accumulation in response to high temperature. Also, Li et al. communicated a transcriptome and flavonoid metabolic study in black and white fruits of *Lycium ruthenicum* Murray. Seven transcription factors (tf) associated with anthocyanin biosynthesis were identified. One of them, *LrAN1b*, a basic helix-loop-helix (bHLH) tf, showed the greatest correlations with anthocyanin accumulation, with no expression in white fruits. Moreover, a new activated anthocyanin MYB tf, *LrAN2-like*, was identified. From these results, it was established that *LrAN1b* and *LrAN2-like* can interact on anthocyanin accumulation in these fruits. It explained the color differences of these fruits, speculating that the white fruit phenotype is due to *LrAN1b* abnormal expression. Furthermore, it was studied how light affects anthocyanin accumulation in *Dendrobium candidum* Wall. ex Lindl. stems, used in traditional medicine and in functional foods (Jia et al.). The results determined that strong light increased anthocyanin content and the expression of genes involved in anthocyanin biosynthesis, upregulating *DcTTG1*, a WD40-repeat tf involved in the expression levels of some key anthocyanin biosynthesis genes (*DcCHS2, DcCHI, DcF3H*, and *DcF3*′*H*). It was also found that *DcTTG1* restored the *Arabidopsis* ttg1-13 mutant phenotype, which is defect in seed coat anthocyanin pigmentation, suggesting a similar function as *Arabidopsis TTG1*. These results will help improve the understanding of the light-induced anthocyanin synthesis and accumulation mechanisms. Moreover, Lim et al. managed to find out why some white-flowered chrysanthemum (*Chrysanthemum morifolium* Ramat.) cultivars produce red rayflorets under natural cultivation conditions, comparing the expression of anthocyanin biosynthetic and tf genes between white ray florets and those that turned red based on cultivation conditions. Significant differences in the bHLH tf gene *CmbHLH2* expression were detected. *CmbHLH2* generated two alternatively spliced transcripts, *CmbHLH2*^*Full*^ and *CmbHLH2*^*Short*^. This second transcript encoded a truncated protein CmbHLH2^Short^ that failed to promote anthocyanin biosynthetic genes not being able to interact with CmMYB6, whereas CmbHLH2^Full^ promoted it when simultaneously expressed with CmMYB6. These results represent an important advance to modulate anthocyanin biosynthesis in chrysanthemum flowers.

Beyond anthocyanins, Sohn et al. provided an exhaustive review on the metabolic engineering process of isoflavone biosynthetic genes in soybean. This study also included a summary about functional roles and health benefits of isoflavones, such as antiosteoporotic properties. In addition, Zhang X. et al. provided remarkable information on the biosynthesis of isoflavone glycosides. Aglycone isoflavones have low water solubility, which limits their therapeutic use; so that, the *O*-glycosylation of isoflavones, catalyzed by *O*-glycosyltransferases (UGTs), would be a way to overcome this problem. So far, studies on isoflavonoid *O*-UGTs were focused on legumes, but this last study is centered on *Iris domestica* (L.) Goldblatt et Mabberley, a non-legume rich in isoflavonoid glycosides. A comparative transcriptome analysis was performed using *I. domestica* seedlings treated with CuCl_2_. Eight new active BcUGTs with broad substrate spectra were obtained. Real-time quantitative PCR results indicated that the transcriptional levels of BcUGTs were induced by Cu^2+^. The different expression levels of BcUGTs in this transcriptome analysis seems to denote that BcUGTs play important roles in responses to abiotic stresses.

When it comes to the absorption, distribution, metabolism and excretion of flavonoids, Sun et al. provided pharmacokinetic information on pinostrobin, an aglycone flavanone found in more than 10 families such as Pinaceae. After its oral administration to male rats, concentrations of pinostrobin and its metabolites in plasma, urine, feces, bile, and tissue homogenates were determined by using ultra-high-performance liquid chromatography coupled with mass spectrometry. These results showed that it was mostly distributed in the gastrointestinal tract, which would explain its antiulcer property. Nevertheless, this compound was widely metabolized *in vivo* and it was able to reach all the organs assessed. This study provided a significant basis for further experiments on the potential biological activity of this compounds.

Regarding pharmacological activities, Lourenço et al. identified a mechanism of action of quercetin 3-*O*-α-*L*-arabinopyranosyl-(1→2)-*O*-α-*L*-rhamnopyranoside as anti-inflammatory compound, by a target *in silico* fishing method. This study also explained the *in vitro* anti-inflammatory activity of this flavonol and that of *Bryophyllum pinnatum* (Lam.) Oken, a flavonol-rich plant commonly used in Madagascar to treat inflammatory some processes. Thus, it was established that this flavonol is a potent PDE4B blocker, highly selective to this enzyme.

Concerning other flavonoid-related compounds activities, this Research Topic also shows the role of xanthohumol (XN), a hop prenylated chalcone, on a high-fat diet (Paraiso et al.). XN supplementation resulted in amelioration of hepatic steatosis and decreased bile acid (BA) concentrations in mice with liver farnesoid X receptor (FXR) deficiency, reducing inflammation and tissue damage. This effect was stronger in male mice. XN induces the androstane, pregnane X and glucocorticoid receptors gene expression in the liver of FXR^Liver−/−^ mice. These data indicated sex-dependent relationship between FXR, lipids and BAs, and that XN improves high-fat diet-induced dysfunctional lipid and BA metabolism via FXR-dependent and independent signaling. Also, the Research Topic includes a wide review recapitulating the mechanisms of action and effects of hydroxysafflor Yellow A (HSYA), a quinochalcone *C*-glycoside extracted from *Carthamus tinctorius* L. flowers, on numerous cardio-cerebrovascular diseases (CCVDs) by considering preclinical studies (Bai et al.). Authors emphasized that, in the future, it will be necessary to perform high-quality clinical trials to support the HSYA application for the control of other CCVDs, and not only of acute ischemic stroke with blood stasis syndrome as up to now.

This Research Topic also shows the mechanism of action of the anticonvulsant glycoside flavone vitexin (apigenin-8-*C*-glucoside), found at plants such as *Passiflora incarnata L*. This compound seems to act mainly via modulation of GABAergic neurotransmission, exhibiting selective protection against tonic-clonic seizures triggered by GABA antagonist (Dias de Oliveira et al.). In addition, experiments carried out in a mouse model demonstrated that the aglycone isoflavone formononetin, the main active component of *Astragalus membranaceus*, improves diabetic renal fibrosis. It inhibited hyperglycemia-induced superoxide overproduction by activating the nuclear factor E2-related factor 2/antioxidant response element signaling pathway and increasing sirtuin-1 protein levels in renal tissue (Zhuang et al.).

Finally, Aneklaphakij et al. contributed with a valuable review about the chemical diversity of seed (poly)phenols in majorly consumed nut species coupled to insights into their biological activities. Furthermore, these authors showed how they used omics-based approaches on nut plant species, presenting an example of the annotation of key genes involved in (poly)phenolic biosynthesis in peanut using comparative genomics.

We hope that the studies shown in this Research Topic contribute to improving the knowledge about flavonoid biosynthesis and their roles in plants, as well as enhancing its use for the prevention/treatment of certain diseases.

## Author Contributions

MG-M participated in the writing of the article and coordinated the work of all the co-authors and designed the figure. MB, CG-V, and PM participated in the manuscript writing. ML-G made the figure and also participated in the writing of the manuscript. All authors contributed to the article and approved the submitted version.

## Conflict of Interest

The authors declare that the research was conducted in the absence of any commercial or financial relationships that could be construed as a potential conflict of interest.

## Publisher's Note

All claims expressed in this article are solely those of the authors and do not necessarily represent those of their affiliated organizations, or those of the publisher, the editors and the reviewers. Any product that may be evaluated in this article, or claim that may be made by its manufacturer, is not guaranteed or endorsed by the publisher.

